# Interactions of working and long-term memory: Evidence from Brown-Peterson and proactive interference effects

**DOI:** 10.3758/s13423-026-02962-z

**Published:** 2026-07-29

**Authors:** Lea M. Bartsch, Eda Mizrak, Klaus Oberauer

**Affiliations:** 1 Univeristy of Zurich, Zurich, Switzerland; 2 University of Oxford & Queen Mary University of London, Oxford, UK

**Keywords:** Working memory, Long-term memory, Secondary task effects, Proactive interference

## Abstract

How do working memory (WM) and episodic long-term memory (eLTM) interact to support immediate memory performance? Their contributions are often intertwined, reflecting a functional overlap that challenges clear-cut distinctions between the two systems. The present study investigates this interaction by assessing the combined effects of two diagnostic manipulations—proactive interference (PI) and secondary task effects (via Brown-Peterson-style filled retention intervals)—on binding tasks across varying set sizes. In Experiment 1**,** participants encoded word-object pairs and completed a four-alternative forced-choice test. Experiment 2 extended this paradigm to the visual domain, using object–color pairings tested via a continuous color reproduction task. In both experiments, PI selectively impaired performance at larger set sizes, consistent with increasing reliance on eLTM under higher memory load. Secondary task effects were strongest at smaller set sizes, reflecting a strong contribution of WM to performance. Secondary task effects impaired performance more strongly under high PI, especially at larger set sizes. This pattern suggests that when interference limits access to eLTM, participants shift reliance to WM—which, in turn, is vulnerable to disruption. These findings support a dynamic view in which WM and eLTM jointly contribute to immediate memory, with their relative involvement modulated by memory load and interference.

When trying to remember a phone number just long enough to dial it or holding a sentence in mind while composing a response, people rely on the ability to store and retrieve information over short delays. Understanding how people solve tasks like these depends on disentangling the respective roles of working memory (WM) and long-term memory (LTM). WM enables the short-term maintenance and manipulation of information relevant for ongoing cognitive tasks and is characterized by limited capacity and rapid accessibility (Baddeley, 1986; Cowan, 2008). In contrast, LTM provides more durable storage with virtually unlimited capacity. Theoretical models diverge on whether WM and LTM are separable systems (e.g., Atkinson & Shiffrin, 1968; Baddeley, 2012), components of a unitary system (e.g., Crowder, 1982; Nairne, 1990, 2002), or overlapping representations, with WM comprising temporarily activated LTM content (Cowan, 1995; Oberauer, 2002).

Many standard WM paradigms challenge participants to briefly maintain a small set of stimuli—usually over a delay of a few seconds—before retrieval. However, WM is not driving performance in these tasks alone (Bartsch & Oberauer, 2023; Oberauer & Bartsch, 2023; Unsworth, 2010; Unsworth et al., 2010). Instead, there is often a contribution from rapidly formed episodic LTM representations (Cowan, 2019; Huebner & Gegenfurtner, 2011). This joint contribution from WM and LTM has both theoretical and methodological implications. Immediate memory tests may not be good measures of WM capacity. If memory performance in immediate recall tasks is supported by both systems, then measures of WM capacity overestimate the actual contribution of WM. Whereas theories of immediate free recall typically assume a substantial contribution from episodic LTM (Grenfell-Essam & Ward, 2012; Ward & Beaman, 2025; Ward et al., 2010), such a contribution has often been less explicit in work on other tests of immediate memory. The present work addresses, this issue by asking when immediate memory for bindings is supported primarily by active maintenance in WM and when it increasingly depends on episodic LTM.

Two complementary tools can be used to disentangle WM and eLTM contributions in immediate memory tasks: proactive interference (PI) and a secondary processing task between encoding and recall. PI is an index of eLTM involvement, whereas vulnerability to a secondary task reflects reliance on WM (see below and Bartsch & Oberuaer 2023). We will review evidence for the selective influence of these two variables next.

## Disentangling contributions of episodic LTM to WM

PI arises when previously learned material interferes with the encoding or retrieval of new information, and it is commonly seen as a hallmark of LTM processes (Crowder, 1976). Whereas LTM-based memory performance is highly susceptible to PI, WM is largely immune to PI (Bartsch et al., 2024; Mizrak & Oberauer, 2020; Oberauer & Greve, 2021; Oberauer et al., 2017). Oberauer et al. (2017) present a more detailed review of the relevant evidence. This functional dissociation allows researchers to estimate the relative contribution of LTM to WM task performance: If PI emerges in a task assumed to reflect WM, it likely indicates a contribution of episodic LTM.

Secondary task effects during the retention interval provide a complementary method for gauging the reliance on WM. This method has been widely used ﻿in the episodic LTM literature, where secondary tasks are ﻿used to displace information from WM to obtain a purer measure of episodic LTM. In the WM literature, the Brown–Peterson paradigm has long been used to investigate forgetting in WM, showing sharp performance declines retention intervals filled with a secondary task, particularly in the first 15–18 s (Brown, 1958; Peterson & Peterson, 1959; Floden et al., 2000). This damage from processing has been observed for verbal (e.g., Jarrold et al., 2010) as well as visual-spatial materials (e.g., Ricker & Cowan, 2010). These effects are taken as evidence that the engagement in a secondary task disrupts maintenance in WM, either by interrupting rehearsal or by introducing interference (see Oberauer et al., 2016; Ricker et al., 2016, for reviews), while leaving LTM largely unaffected. Consequently, when performance suffers as a function of secondary task-filled intervals, it indicates reliance on WM. Conversely, if performance is resilient to secondary task effects this suggests a shift toward contributions from LTM.

These two methods have been recently used to establish a double dissociation between the contributions of WM and LTM to an immediate memory task requiring the maintenance of bindings: LTM strongly contributed to performance at set sizes larger than 3, whereas performance at set size 2 was driven by WM (Bartsch & Oberauer, 2023). Specifically, PI emerged only at larger set sizes, whereas a secondary task during retention only affected performance at smaller set sizes, suggesting a more WM-dependent performance.

## The interaction of secondary-task Effects and PI

If episodic LTM contributes more strongly under conditions that strain WM—such as high set sizes or secondary task processing—it follows that PI effects should be more pronounced if there is a secondary task that reduces WM availability and hence leads to more reliance on LTM. Conversely, when LTM has been compromised by PI, motivating stronger reliance on WM, the effect of a secondary task in the retention interval should be magnified. Initial evidence for this interaction comes from studies showing that the effects of secondary task-filled retention intervals are much attenuated when PI is absent, as, for example, on a participant’s first trial in a Brown–Peterson memory task (e.g., Keppel & Underwood, 1962; Loess, 1964; Meudell, 1977).

## The present study

Building on this prior work, the current study investigates the contribution of episodic LTM to two WM binding tasks at varying set sizes. To disentangle WM and LTM contributions, we assessed the interaction of secondary task effects (i.e., the Brown–Peterson effect) and PI on immediate memory for bindings.

If LTM is recruited more heavily when WM is taxed, we expect PI effects to emerge primarily at larger set sizes without secondary task, and already at smaller set sizes when WM is disrupted by a secondary task. Conversely, we expect secondary task effects to be particularly pronounced at smaller set sizes but also emerge at larger set sizes under high-PI conditions. With high PI, access to episodic LTM is impaired by interference from previously encoded material, limiting its usefulness for supporting performance. As a result, participants must rely more on WM representations, which are vulnerable to disruption by the secondary task.

Beyond testing these predicted interactions, we used state-trace analysis to evaluate whether the pattern of results is compatible with a single latent memory dimension or instead requires separable contributions of WM and episodic LTM. A double dissociation is suggestive of distinct processes, but it does not by itself rule out a unitary account in which both dependent measures are monotonic functions of a common underlying variable. State-trace analysis provides a stronger test of this possibility by examining whether performance at low and high set sizes can be ordered along a single monotonic function across the experimental conditions (Newell & Dunn, 2008). If performance at both set-size ranges reflects one common memory-strength dimension, the state trace should be monotonic. If PI and secondary-task processing affect separable WM and episodic LTM contributions, however, the state trace may deviate from monotonicity. As a complementary test of a unitary account, we also examined whether the SIMPLE model (Brown et al., 2007), a model in which memory performance is determined by temporal distinctiveness rather than separate WM and LTM systems, can reproduce the observed pattern.

In Experiment 1, we tested these predictions using a verbal binding task in which we manipulated set size, PI, and whether there was a secondary task-filled RI. Experiment 2 tested the generalizability of our findings using a different domain and testing format which tested memory for object–color pairings using a continuous color-reproduction task.

## Experiment 1

### Method

#### Participants

We collected data from 50 participants (*M*_age_ = 23.40 years) in the lab. All participants’ first language was German or Swiss-German, and they reported normal or corrected-to-normal vision. Participants signed an informed consent form prior to the study and were debriefed at the end. We chose the sample size for this, and the second experiment because it was sufficient to detect the effects of interest in a similar study (see Bartsch & Oberauer, 2023). The use of Bayesian statistics means that the sample size could have been increased in case of ambiguous evidence (Rouder, 2014). This was the case, so that after initial collection of 29 participants, we increased sample size to *N* = 50.

#### Materials and procedure

**Fig. 1:**
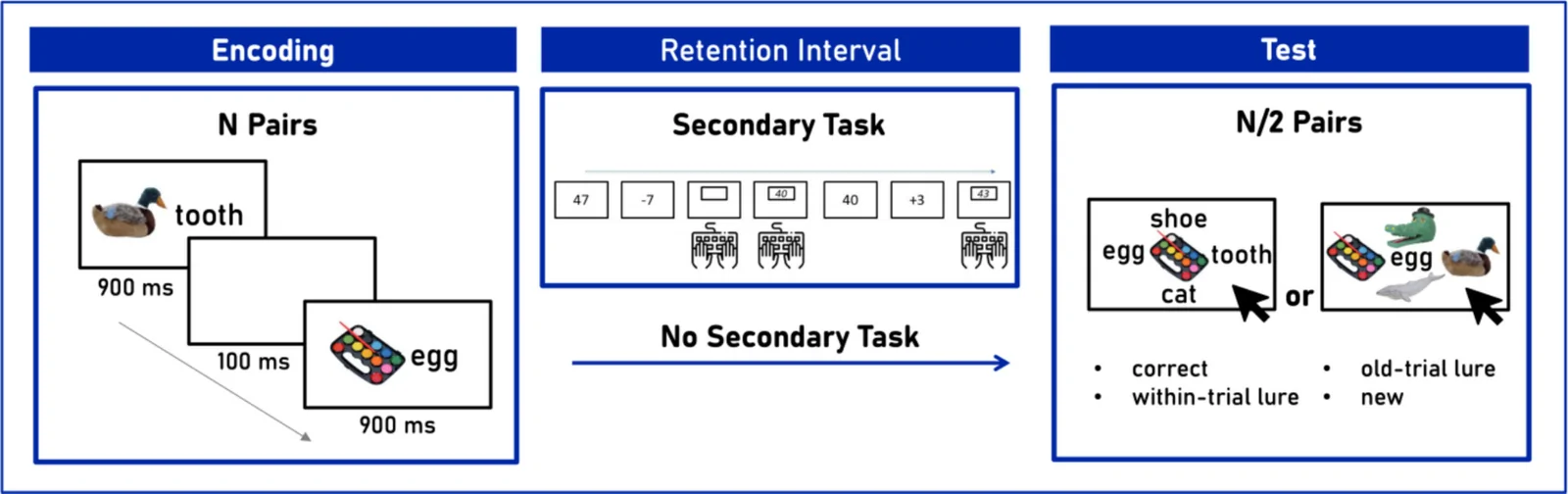
Illustration of the paradigm of Experiment 1. Subjects were sequentially shown a list of word–picture pairs at varying set sizes. In half of the trials a filled retention interval of 15 s was implemented prior to the test. In the other half of the trials the test followed immediately. Immediate memory was tested for half of the pairs in random order, by either cueing with the picture or word. Subjects had to choose between the four response options, comprising the correct item, a same-trial lure, an old-trial lure, and a new item. (Color figure online)

Figure 1 provides an overview of the general procedure of Experiment 1. The paradigm was based on a previous study and aimed to measure working memory for bindings (see Bartsch & Oberauer, 2023). We sequentially presented arbitrary word–picture pairs at set sizes 2, 3, 4, and 6. Words were drawn from a pool of 1,198 neutral words from the Berlin Affective Word List (BAWL-R; Vo et al., 2009), with a minimum length of two and a maximum length of 10 letters. The words had a mean frequency per million of 59.33. Pictures were drawn from a pool of 2,400 photographs of real-world objects (Brady et al., 2008). The experiment involved two sessions of 1.5-h comprising 160 trials each, with 20 trials per cell of the design (set size × PI × secondary task). The PI conditions (PI vs. no PI) were varied between sessions, and the cue (word vs. picture cue) was held constant within participants (half the participants were always cued with words, and the other half with pictures). The secondary task varied from trial to trial (secondary task vs. none), with half of the trials being followed by a 15-s retention interval (RI) filled with a math task. This distractor task consisted of a continuous series of simple arithmetic problems. Each trial began with a number between 9 and 90 (e.g., 63), followed by an operation (e.g., + 6), and participants were asked to calculate the result of the operation and enter their response in a box and press enter (e.g., 69). The next operation (e.g., − 4) was then to be applied to their previous response (e.g., 69 − 4 = 65) and the new result was entered in a new box, and this process was repeated for 15 s. Each operation was presented for 1 000 ms, and the response time was unlimited. After 15 s elapsed, a blank screen appeared for 1,000 ms before the recall screen was presented.

The order of sessions was counterbalanced between participants. In the PI session, the stimuli were repeatedly used across trials, as they were drawn from sets of 12 words and 12 pictures with replacement. Within each trial no word or picture was repeated. In the low-PI session, the stimuli were drawn from the entire pool of 1,198 words and 2,400 pictures after removing the 12 words and 12 pictures used for the high-PI session. Stimuli were sampled without replacement, ensuring that no stimulus ever repeated across trials.

Each pair was presented for 900 ms with a 100-ms interstimulus interval. After a 1,000-ms retention interval (no secondary task condition) or the 15-s RI (secondary task condition), immediate memory was tested for a random half of the presented pairs in random order with a four-alternative forced-choice (4-AFC) procedure. In the odd-numbered set size 3 trials, only one of the three pairs was tested. Half of the subjects were cued with the word of a pair and were to choose from four pictures displayed around it by clicking on the option they thought to have been presented together with the word at encoding (word-cue condition). The other half of the subjects were cued with a picture and were to choose from four words (picture-cue condition). The four options always comprised the correct item, a same-trial lure (an element from another pair of the same trial), a lure from a previous trial (old intrusion lure; selected from any randomly chosen prior trial) and a new item (new lure). We made sure that the lures from a previous trial were chosen such that they did not repeat in the current trial. The response options were assigned at random to their location on the screen.

As in previous work, this test was designed to distinguish item and binding memory: Rejection of the within-trial lure requires memory for the correct binding between the respectively cued picture (or word) and the target word (or picture). If subjects do not have intact binding memory but accurate item memory, they would choose between the target and within-trial lure with equal probability. Similarly, rejection of the old-trial lure requires memory for the correct items of the current trial. Only if subjects have no memory at all would they guess between all four options with equal probability.

The within-trial lure was always drawn from a pair presented immediately before or after the target pair to control for increasing context changes across the pairs within a trial at larger set sizes. The reason for only testing half of the presented pairs was that we wanted to avoid repeating any response option across tests to minimize dependencies between responses in the same trial. Therefore, the within -trial lures were drawn from the not-tested pairs.

#### Data analysis

We analyzed the data of Experiment 1 using a Bayesian hierarchical logistic regression predicting correct or incorrect responses in the 4-AFC test by memory set size, proactive interference and secondary task. The dependent variable was the number of correct responses out of the number of retrievals for each cell of the design. Therefore, we assumed a binomial data distribution predicted by a linear model through a logit link function (i.e., a logistic regression). Correct responses were defined as choosing the target item from the alternatives. The fixed effects were proactive interference, secondary task, and set size, and the latter was included in the model as a continuous predictor. The model included a random intercept, and random slopes of set size, secondary task, and proactive interference.

In case we found any evidence in favor of one of the fixed effects, we tested whether it was driven by reduced memory for bindings in WM, which would become manifest in an increase in probability to choose the within-trial lure above all the remaining options—or whether it affected the probability of choosing old trial lures. We implemented this in a Bayesian hierarchical logistic regression model predicting the probability of errors in the 4-AFC test separately for each type of error (within-trial intrusion, old intrusion lure, and new lure).

It is notable that the four dependent variables are not independent, because the number of responses for each of the four categories (correct, within-trial intrusion, old intrusion lure, and new lure) sum up to the number of tests. Therefore, one of these analyses is redundant. We nevertheless report all four to present the evidence for the fixed effects on each category of responses in the most transparent way.

The model was implemented in the R package *brms* (Bürkner, 2017, 2018). The regression coefficients were given moderately informative normal priors with a standard deviation of 1. This defines a default prior analogous to that proposed by Rouder et al. (2012) for the general linear model. We used a Markov chain Monte Carlo (MCMC) algorithm (implemented in *Stan*; Carpenter et al., 2017) that estimates the posteriors by sampling parameter values proportional to the product of prior and likelihood. These samples are generated through four independent Markov chains, with 2,000 warmup samples each, followed by 12,500 samples drawn from the posterior distribution which are retained for analysis (50,000 retained in total). Following Gelman and colleagues (2013), we confirmed that the four chains converged to the same posterior distribution by verifying that the R-hat statistic—reflecting the ratio of between-chain variance to within-chain variance—was < 1.02 for all parameters, and we visually inspected the chains for convergence. Finally, we estimated Bayes factors using the Savage–Dickey density ratio (Wagenmakers et al., 2010) between the estimated posterior distribution and the exact prior distribution.

### Results

#### Accuracy

**Fig. 2:**
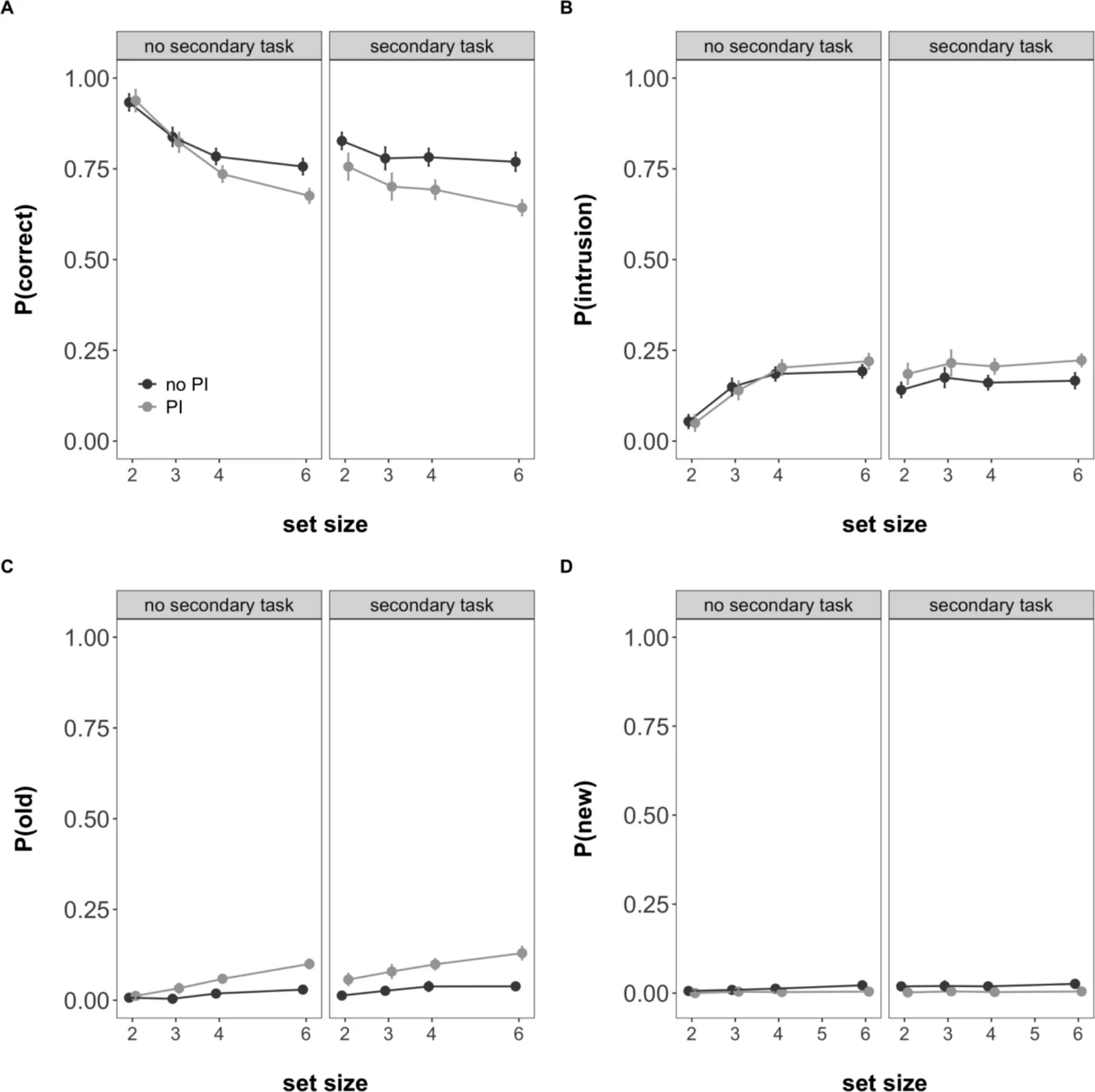
Immediate memory performance in Experiment 1. **A** Probability of choosing the correct item across set sizes. **B**, **C**, and **D** Probability of choosing the same-trial lure from the same trial, the old-trial lure from a previous trial, and a new item, respectively. The subpanels represent the conditions with vs. without a secondary task following encoding. The colors distinguish the no PI from the PI session. Error bars represent the within subject confidence interval. Chance level is 0.25

Figure 2A shows the mean probability of choosing the correct response option across the conditions. We predicted (1) an interaction of set size with PI, reflecting a PI effect only at larger set sizes; (2) an interaction of set size with secondary task, reflecting a secondary task effect only at smaller set sizes, and (3) an interaction of secondary task with PI, showing more PI in the presence of secondary task. The Bayesian hierarchical logistic regression revealed evidence for all the predicted two-way interaction effects (see Table 1 for all BFs) as well as evidence for all the main effects. As expected, there was a higher probability of choosing the correct item at smaller compared to larger set sizes, and without secondary task than with secondary task.

**Table 1:** Bayes factors of all main effects and interactions of Experiment 1

Effect	BF_10_	BF_01_
Set Size	**1.68 × 10**^**12**^	–
Proactive Interference (PI)	**9.55**	**–**
Secondary task	**5.74 × 10**^**10**^	–
Set size × PI	**11.37**	–
Set size × Secondary task	**2.02 × 10**^**9**^	–
PI × Secondary task	**3.01**	–
Set size × PI × secondary task	0.04	**26.36**

BFs > 3 in favor of or against an effect are printed in **bold**

#### Effects of PI

Follow-up analyses revealed that without any secondary task in the retention interval, PI only (negatively) affected the probability of choosing the correct option for set sizes 4 and 6 (BF_10_ = 33.14, and BF_10_ = 2.53** × **10^**3**^, respectively), but had no detrimental effect at set sizes 2, and 3 (BF_01_ = 4.98, and BF_01_ = 6.85, respectively).

When a secondary task was introduced, PI negatively affected performance across all set sizes (SS2: BF_10_ = 40.17; SS3: BF_10_ = 77.00; SS4: BF_10_ = 8.27** × **10^**3**^; SS6: BF_10_ = 1.03** × **10^**6**^). These results indicate that without a secondary task, performance at low set sizes relies on WM, with PI not affecting performance. As a secondary task is inserted in the retention interval, performance is at least in part dependent on LTM at all set sizes, leading to credible PI effects.

#### Effects of secondary task

In conditions without PI, the secondary task cost was credible only at set size 2 (BF_10_ = 1.34 × 10^6^). Evidence for or against a secondary-task effect was ambiguous at set size 3 (SS3: BF_10_ = 1.40), and secondary task did not affect any of the other set sizes (SS4: BF_01_ = 10.64 and SS6: BF_01_ = 6.25). Yet, under PI, secondary task effects occurred up until set size 3 (SS2: BF_10_ = 1.21 × 10^10^; SS3: BF_10_ = 9.59 × 10^3^). At larger set sizes evidence for the secondary task effect became ambiguous (SS4: BF_01_ = 2.02 and SS6: BF_01_ = 1.21). These results corroborate the above findings, showing that in conditions with low PI, LTM could still compensate the detrimental effect of the secondary task on WM traces, leading to secondary-task costs being confined to the lowest set size. Under PI, when the contribution of LTM is less helpful, the secondary task also disrupts performance at somewhat larger set sizes.

#### Interaction of PI and secondary task

The effects of PI and secondary task on performance were qualified by a credible interaction. Follow-up analyses revealed that whereas under PI there was strong evidence for an overall effect of the secondary task (BF_10_ = 1.51 × 10^4^), there was evidence against such an effect in case there was no PI (BF_01_ = 14.29). As predicted, the secondary task increased reliance on episodic LTM, leading to stronger PI. Under high PI, performance relied more on WM, resulting in greater sensitivity to the secondary task compared to low-PI conditions.

#### Error types

Our second question was whether the modulation of the set size effect by the secondary task was driven by reduced memory for bindings, which would become manifest in an increase in probability to choose the same-trial lure above all the remaining options reflecting WM contribution. Further, we expected the effects of PI to manifest in an increase in probability to choose the old intrusion lures.

The BFs of all effects with each type of error can be found in Table 2. We found that ﻿the probability of choosing the same-trial lure was the only error type showing an interaction between set size and any of the other factors, specifically with the secondary task. Here, follow-up analyses showed that ﻿only for set sizes 2 and 3 there was an effect of the secondary task on the probability of choosing the same-trial lure (BF_10_ = 7.00 × 10^10^and BF_10_ = 6.31, respectively), whereas there was ambiguous to substantial evidence against this effect at set sizes larger than 3 (SS4: BF_01_ = 2.69 and SS6 BF_01_ = 5)—replicating previous research (Bartsch & Oberauer, 2023).

**Table 2:** Bayes factors (BF_10_) for the effects of the independent variables on different error types in Experiment 1

Effect	Same-trial lures	Old-intrusion lures	New lures
Set size	**1.20 × 10**^**9**^	**2.88 × 10**^**4**^	**1.59 × 10**^**3**^
Proactive interference (PI)	0.14	**2.37 × 10**^**4**^	**743**
Secondary task	**236**	**665**	**8.67**
Set size × PI	0.16	0.20	0.27
Set size × Secondary task	**2.11 × 10**^**4**^	2.04	1.61
PI × Secondary task	2.88	0.34	1.73
Set size × PI × Secondary task	0.09	0.15	0.29

BFs > 3 in favor of an effect are printed in **bold**

As expected, PI did not affect the probability of same-trial lures (i.e., binding errors), but instead led to more old intrusion errors as well as a reduced probability to choose new lures (see Table 2 and Fig. 2C–D).

### Discussion

We replicated prior findings showing that LTM contributes more strongly at larger set sizes, as PI effects were limited to set sizes 4 and 6. The PI effect became manifest primarily as an increase in erroneous selections of lures from preceding trials, as is expected from PI between trials (Gardiner et al., 1972). One potential worry is that performance at set size 2 was close to ceiling, potentially preventing an effect of PI from becoming manifest. The fact that the 95% confidence intervals are safely away from ceiling speaks against this possibility: There is room for performance to measurably improve. Moreover, the compression of experimental effects close to ceiling on the proportion-correct scale is accounted for by our logistic model.

The new finding is the interaction between secondary task processing and PI: In case a secondary task was present in the retention interval, PI effects also emerged at smaller set sizes. In the high-PI condition, the secondary task had a larger effect, because performance had to rely more on WM, which is vulnerable to a secondary task. Taken together our results speak for the involvement of episodic LTM at larger set sizes and in case of a secondary task during the retention interval. These are the conditions under which WM cannot cope with the task demand on its own, and therefore the memory system must rely on episodic LTM.

## Experiment 2

In Experiment 2 we tested whether our findings generalized to other information domains by measuring memory for visual bindings, and to other types of testing by using a continuous reproduction task instead of a 4-AFC test.

### Method

#### Participants

We collected data from 40 participants (*M*_age_ = 23.40 years) in the lab. All participants’ first language was German or Swiss-German, and they reported normal or corrected-to-normal vision. Participants signed an informed consent form prior to the study and were debriefed at the end.

#### Materials and procedure

**Fig. 3:**
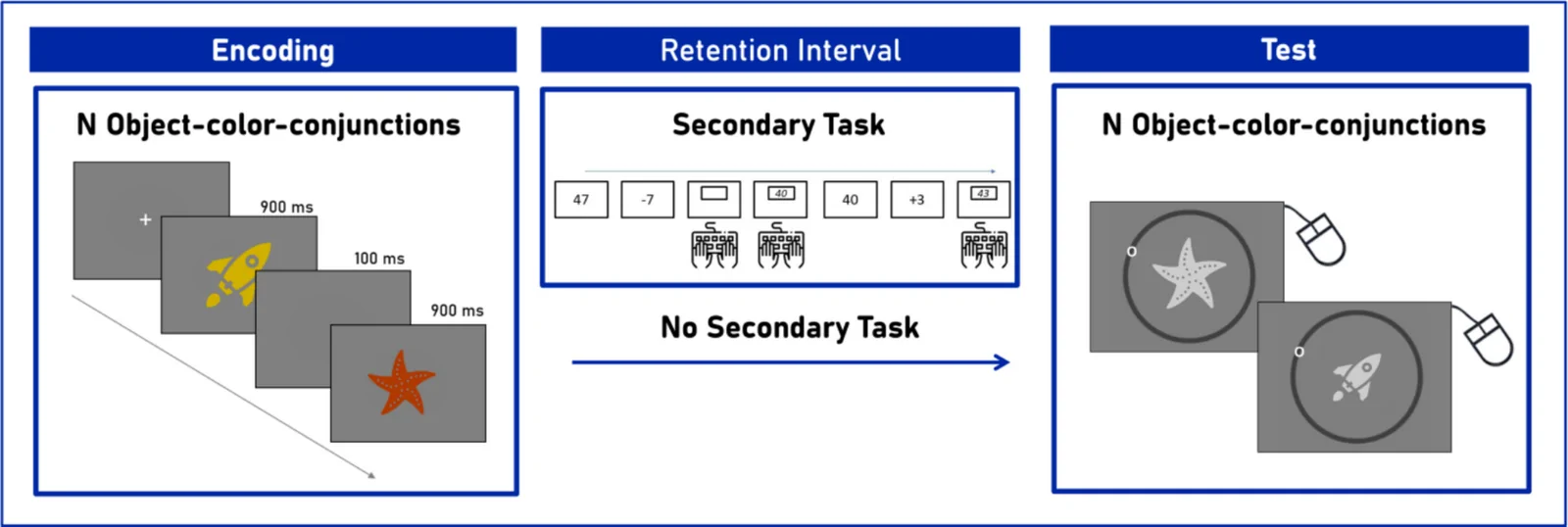
Illustration of the paradigm of Experiment 2. Subjects were sequentially shown a list of object–color conjunctions at varying set sizes. In half of the trials a filled retention interval of 15 s was inserted prior to the test. In the other half of the trials the test followed immediately. Immediate memory was tested for all of the conjunctions in random order, by cueing with the object colored in gray. Subjects had to move the mouse on a gray-wheel to choose the color that was associated with the object. The object continuously changed color according to the position of the mouse on the gray-wheel. (Color figure online)

Experiment 2 was based on the procedure of Experiment 1, except for the following changes: Here, we sequentially presented arbitrary object–color conjunctions at set sizes 1, 2, 3, 4, 6 and 8 (see Fig. 3). Silhouettes of objects were filled with one of 360 colors selected from a continuous color wheel defined in the CIELAB color space with L = 70, a = 20, and b = 38, and radius = 60. The silhouettes were obtained from the set used by Sutterer and Awh (2016).

Immediate memory was tested using a color reproduction task: The silhouette of one of the objects of the current trial appeared on the screen center for 1 s, followed by the presentation of a gray wheel surrounding it. Participants moved the mouse to a location in the gray wheel to reveal the color at that location: While moving the mouse cursor along the wheel, the object was filled with the color at the current mouse position. When participants were satisfied with the selected color, they pressed the mouse button to confirm their response. All objects were tested in this way in a random order.

As Experiment 1, the experiment involved two-sessions of 1.5 h, but here they comprised 144 trials each, with 24 trials per cell of the design (set size × PI × secondary task). The PI conditions (PI vs. no PI) were varied between sessions. Again, secondary task varied from trial to trial (secondary task vs. none), with half of the trials being followed by a 15-s retention interval (RI) filled with a math task. The order of sessions was counterbalanced between participants. In the PI session, the stimuli were drawn from sets of 12 objects and in each trial the silhouettes were drawn from this pool without replacement, so that each stimulus was used only once per trial. In the low-PI session, the objects were drawn from the entire pool of 385 objects, with objects being repeated a maximum of 2 times (only half of the stimuli had to be repeated, and the objects were resampled only after each stimulus had been shown once).

#### Data analysis

The data were analyzed equivalently to Experiment 1, only that here, the dependent variable was the mean recall error computed as the absolute difference between the presented color and participants’ response. This measure can range from 0° (the correct color was chosen) to 180° (a color on the opposite side of the color wheel was chosen). Therefore, we assumed a Gaussian distribution predicted by a linear model through an identity link function. We *z*-standardized the dependent variable. For regression coefficients we used moderately informative Cauchy priors with a scale of .5. This defines a default prior analogous to that proposed by Rouder et al. (2012) for the general linear model.

### Results

#### Mean recall error

**Fig. 4:**
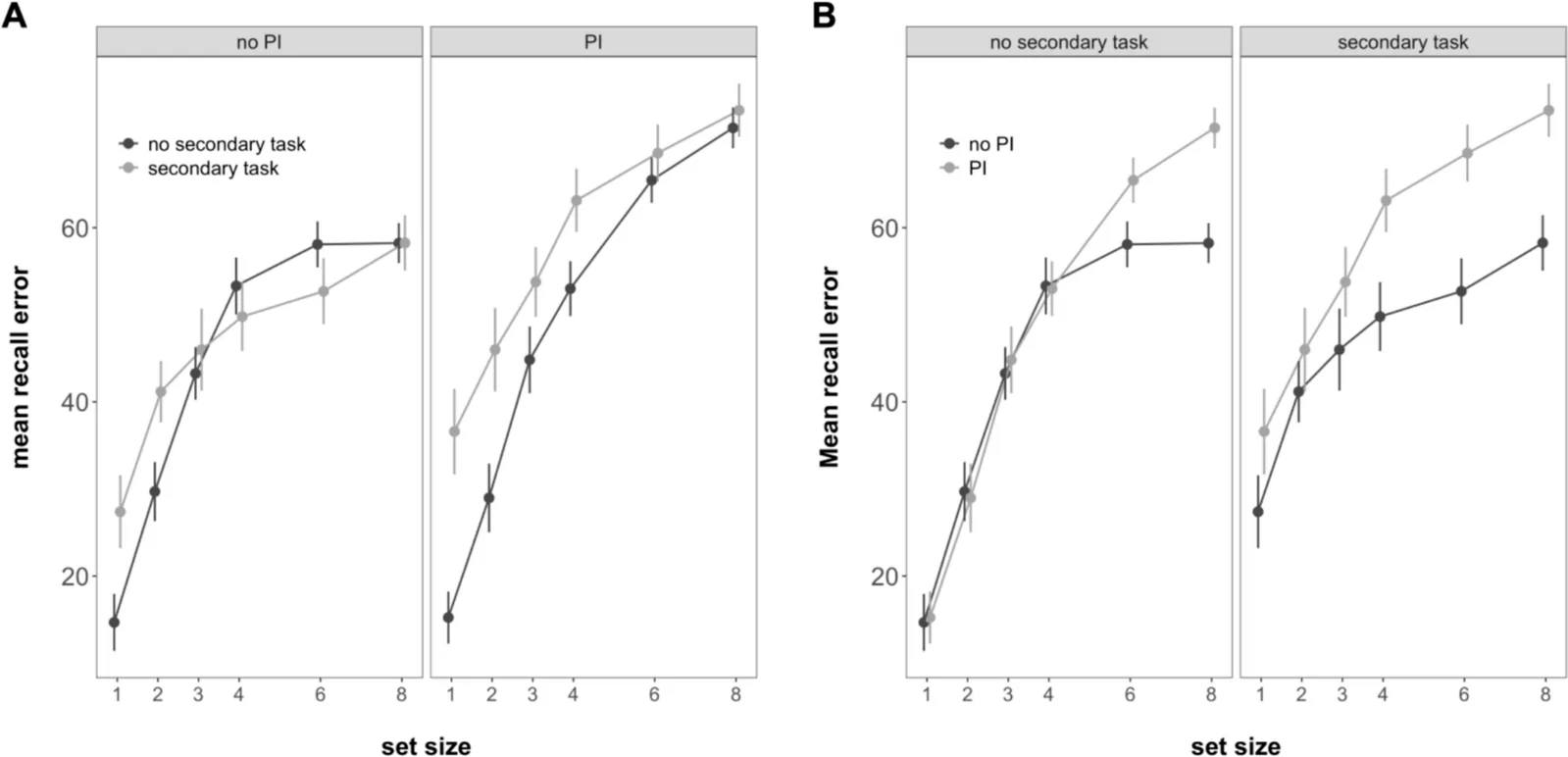
Mean recall error in the immediate *m*emory test of Experiment 2. **A** *F*acets show the PI vs. no PI session and color distinguishes the conditions with vs. without a secondary task prior to recall. **B** Facets show the conditions with vs. without a secondary task prior to recall and the colors distinguishes the PI vs. no PI session. Error bars represent within-subject confidence intervals

Figure 4 shows the mean recall error across set sizes in conditions of high PI and low PI as well as with and without a secondary task in the retention interval. Our first question was whether we replicate the pattern of interaction of the secondary task and PI manifesting at different set sizes, which we found in Experiment 1. Specifically, we predicted that (1) the secondary task impairs performance only at low set sizes, when the task is handled by WM only; (2) PI impairs performance primarily at higher set sizes, when episodic LTM is recruited to assist; (3) PI should interact with secondary task processing: In the presence of a secondary task, performance relies more strongly on episodic LTM, even at lower set sizes, so that PI has a larger effect than in the absence of a secondary task. Conversely, in the presence of high PI, performance must rely more on WM, so that it becomes more vulnerable to a secondary task than when PI is low and episodic LTM can help out. The Bayesian hierarchical linear regression revealed substantial evidence for all the predicted two-way interactions (see Table 3 for all BFs) as well as strong evidence for the main effects of set size and secondary task. Overall, there was lower mean error at smaller compared with larger set sizes, and without a secondary task than with a secondary task. Further, there was credible evidence for the main effect of PI, with better performance in the low-PI condition. Overall, we replicated all effects of Experiment 1.

**Table 3:** Bayes factors of all main effects and interactions of Experiment 2

Effect	BF_10_	BF_01_
Set size	**1.14 × 10**^**37**^	–
Proactive interference (PI)	**43.66**	–
Secondary task	**6.59 × 10**^**10**^	–
Set size × PI	**1.55 × 10**^**3**^	–
Set size × Secondary task	**8.93 × 10**^**5**^	–
PI × Secondary task	**125**	–
Set size × PI × Secondary task	0.04	**24.39**

BFs > 3 in favor of or against an effect are printed in **bold**

#### Effects of PI

Follow-up analyses revealed that PI only (negatively) affected memory performance at set sizes larger than 3 (SS4: BF_10_ = 11.86, SS6: BF_10_ = 7.07** × **10^5^, SS8: BF_10_ = 7.45** × **10^7^), whereas there was anecdotal to substantial evidence that performance was unaffected at smaller set sizes (SS1: BF_01_ = 2.99, SS2: BF_01_ = 11.63, SS3: BF_01_ = 2.00). These findings generalized those of Experiment 1 and provide ﻿evidence for the greater involvement of episodic LTM at larger set sizes for visual bindings.

#### Effects of the secondary task

Here, we asked whether the secondary task affected memory performance differentially across set sizes. As shown in Table 3 there was evidence for an interaction between secondary task and set size. Follow-up analyses revealed that the secondary task only (negatively) affected the performance for set sizes 1 and 2, (BF_10_ = 8.13** × **10^6^ and BF_10_ = 8.77** × **10^5^, respectively), but had no detrimental effect at set sizes 4, 6, and 8 (BF_01_ = 4.41, BF_01_ = 18.87, BF_01_ = 16.95, respectively). Evidence for an effect at set size 3 was anecdotal (BF_10_ = 1.58) The finding that a secondary task -filled delay impairs performance for small but not larger set sizes suggests that WM plays a greater role at smaller set sizes. At larger set sizes, performance appears to rely primarily—or exclusively—on LTM, making it less susceptible to interference during the retention interval.

#### Interaction of PI and the secondary task effect

The effects of PI and the secondary task on performance were qualified by a credible interaction (see Table 3). Follow-up analyses revealed that whereas under PI there was strong evidence for an overall effect of the secondary task (BF_10_ = 4.78** × **10^3^), there was evidence against such an effect in case there was no PI (BF_01_ = 3.70). These findings support our prediction that the secondary task shifts reliance toward episodic LTM, even at smaller set sizes, thereby increasing the impact of proactive interference. Conversely, when PI is high and access to LTM becomes unreliable, performance depends more heavily on WM, making it more susceptible to disruption from a secondary task compared to conditions with low PI, where LTM can still support memory.

### Discussion

Experiment 2 replicated the double dissociation found in Experiment 1, showing distinct contributions of WM and LTM across set sizes in object–color conjunctions. Specifically, PI selectively harmed performance at larger set sizes—replicating Oberauer and Awh (2022)—whereas a secondary task during the retention interval selectively impaired recall at smaller set sizes. Furthermore, the interaction of the secondary task and PI indicates that in case of low PI, performance can rely strongly on LTM and a secondary task in the retention interval therefore does not harm performance. Only when PI makes contributions from LTM less reliable a secondary task negatively affects performance, because in this condition WM must take over a larger share in what drives performance.

#### State-trace analysis and comparison with a unitary memory model

A double dissociation is often viewed as strong evidence for two distinct processes, but this conclusion can be misleading under some conditions. State-trace analysis provides a more robust way to evaluate process separability (Newell & Dunn, 2008). The method requires two dependent measures that index the two assumed processes, respectively—in our case, performance at small set sizes, assumed to rely mainly on WM, and performance at larger set sizes, assumed to draw more on episodic LTM. Low set sizes were defined as set sizes ≤ 3, and high set sizes as set sizes ≥ 4. This cut-off was chosen because estimates of WM capacity, when expressed as the number of chunks that can be maintained, often lie around three to four (Adam et al., 2017; Cowan, 2000; Zhao & Vogel, 2023).

**Fig. 5:**
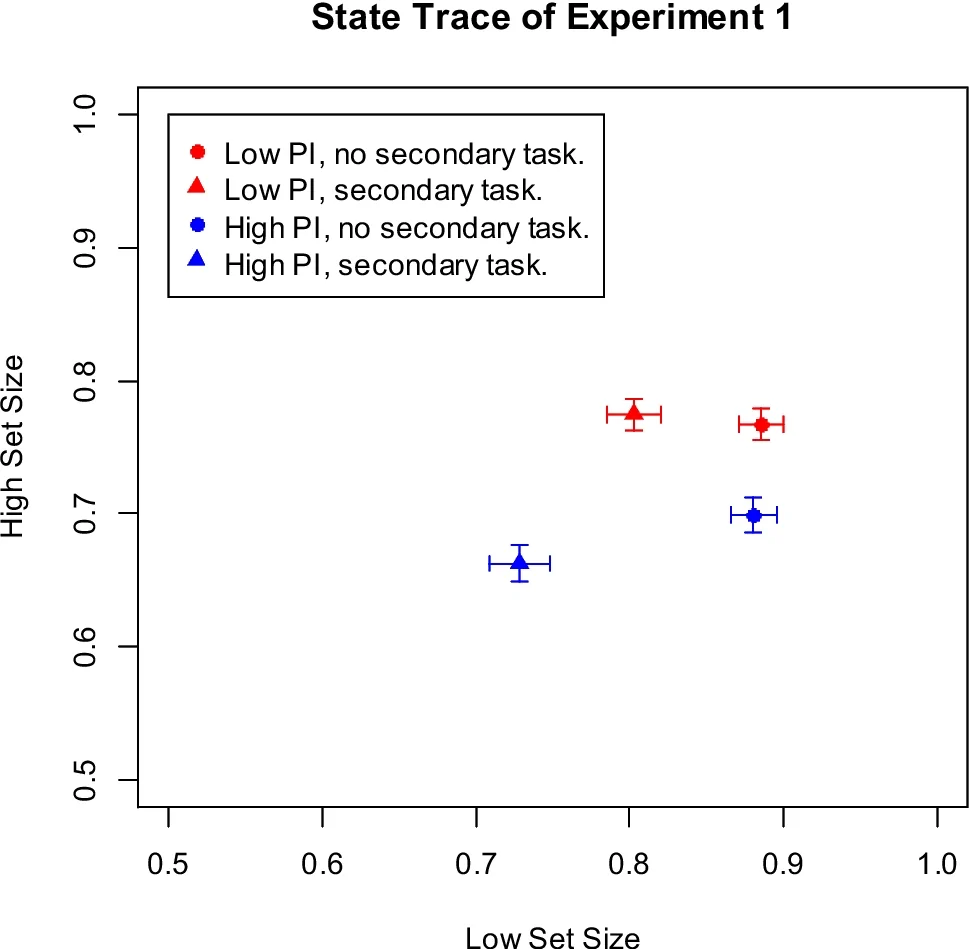

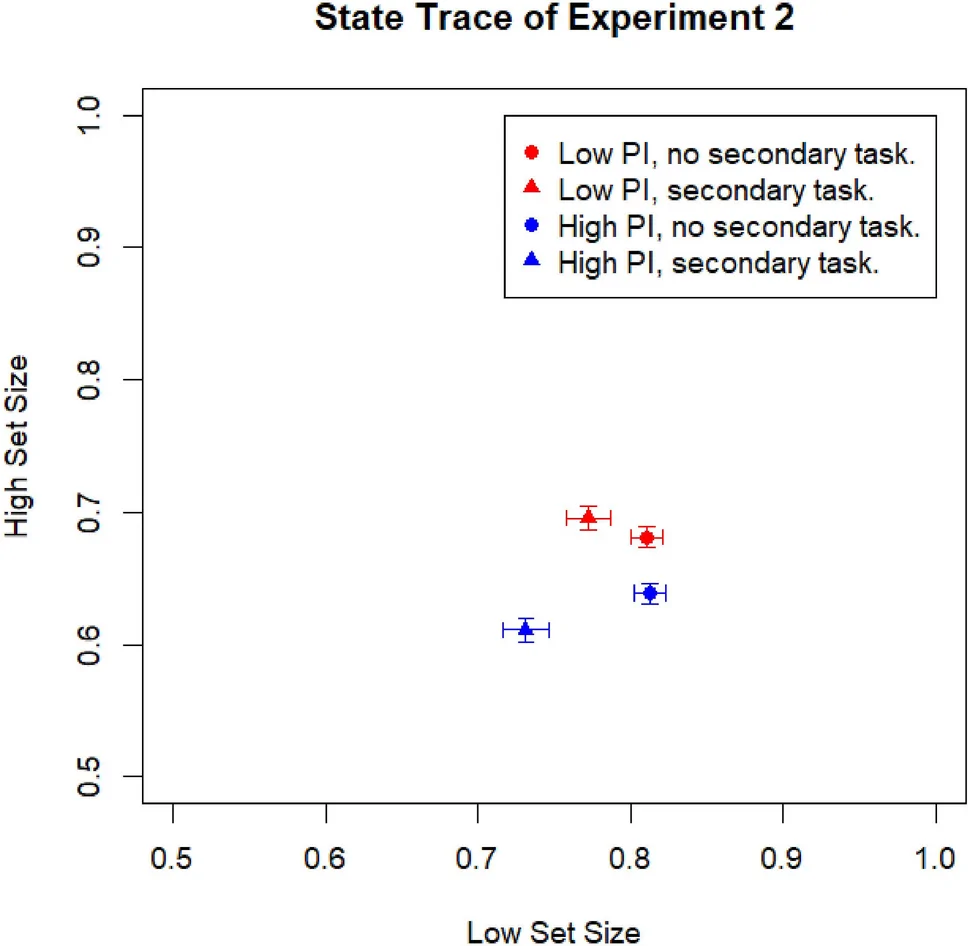
State-trace plots of mean accuracy at high set sizes (≥ 4) against mean accuracy at low set sizes (≤ 3). In Experiments 1 and 2. Error bars are 95% within-subjects confidence intervals. (Color figure online)

The method also requires at least two independent variables whose effects on both measures can be compared; here, these are the PI manipulation and the secondary task-filled retention interval. The state trace plots the relation between small-set-size and large-set-size performance across all conditions (Fig. 5).

For Experiment 1, the state-trace analysis was based on mean accuracy, with higher values reflecting better performance. For Experiment 2, we transformed mean recall error so that larger values likewise reflected better performance [i.e., accuracy = 1 − (error/180°)]. Thus, in both experiments, values further along each axis indicate better memory performance.

We consider two alternative theoretical scenarios: If a single latent dimension (e.g., memory strength in a unitary memory system) drives performance at all set sizes, then all points should lie along one monotonic function. If instead WM and episodic LTM reflect separate latent dimensions, each affected differently by the independent variables, the state trace is not constrained to a single monotonic curve. The pattern observed Fig. 5 aligns with this two-dimensional account: No monotonic curve can be drawn that connects the four points within their confidence intervals.

We formally tested whether the observed state-trace patterns were compatible with a single monotonic latent dimension using conjoint monotone regression following Dunn and Kalish (2018). For both experiments, the analyses indicated significant deviations from monotonicity (Experiment 1: SSE = 20.77, *p* < 0.001; Experiment 2: SSE = 10.99, *p* = 0.002). Thus, the state-trace patterns cannot be adequately described by a single monotonic function relating low-set-size and high-set-size performance across conditions. These results provide formal support for the distinction between WM and episodic LTM.

**Fig. 6:**
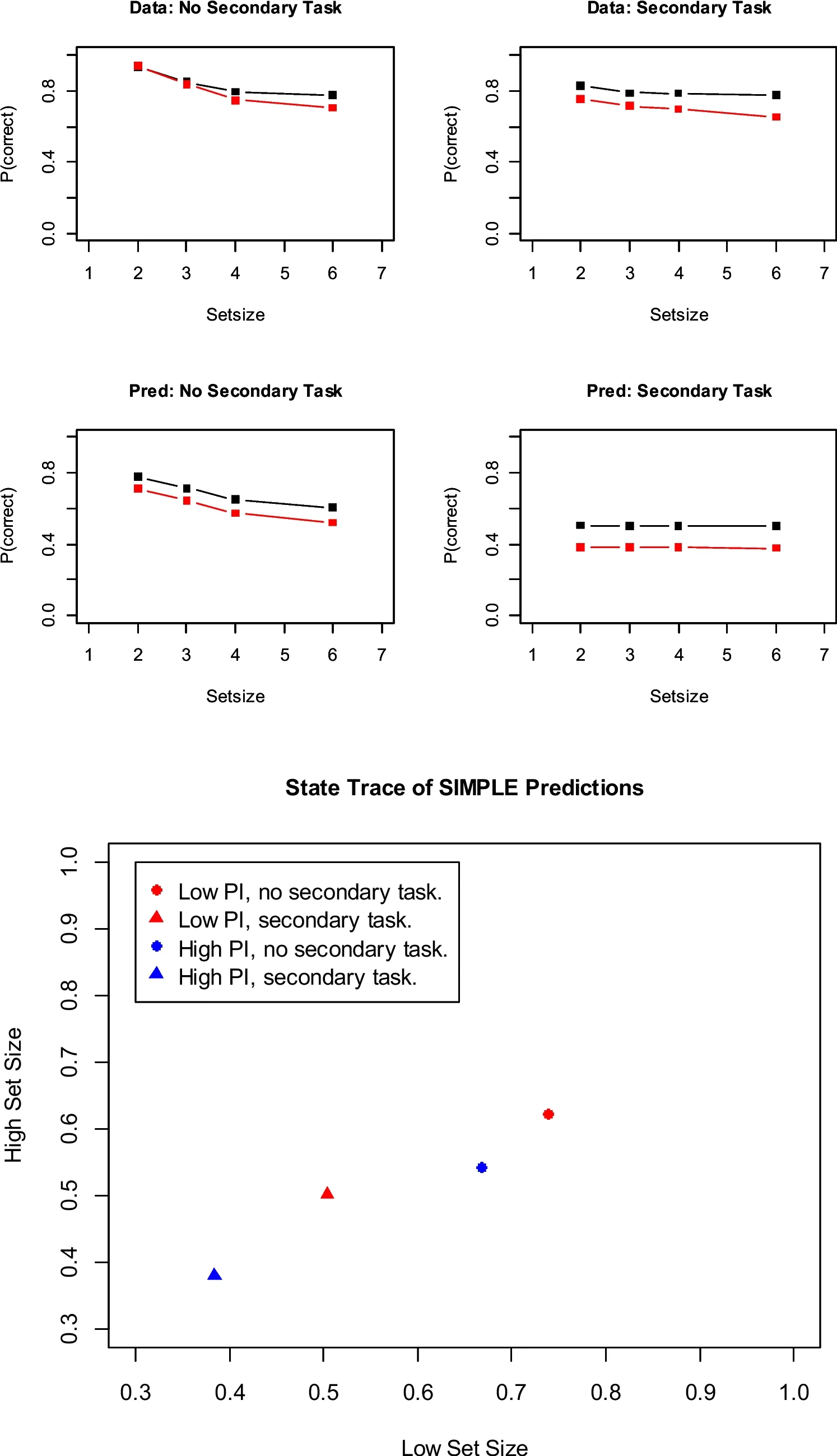
Data and model predictions of SIMPLE. The effects of set size, PI, and secondary-task processing predicted with the best-fitting parameter values. (Color figure online)

As a second test of the viability of a unitary memory theory we investigated whether the SIMPLE model (Brown et al., 2007) can explain our results. In SIMPLE there is no distinction between WM and LTM. The chance of retrieving a memory representation depends on its temporal distinctiveness, which decreases with longer retention intervals. As such, SIMPLE can explain why an extended filled retention interval impairs performance. With lower temporal distinctiveness between trials, PI becomes more likely, and therefore SIMPLE can also explain why PI increases with a longer retention interval. We fit SIMPLE to the data of Experiment 1. The upper four panels of Fig. 6 show the effects of set size, PI, and secondary-task processing predicted with the best-fitting parameter values. The model accounts for the main effects but fails to account for the interaction of set size with secondary-task interference. This is because SIMPLE explains the secondary-task cost through the reduced temporal distinctiveness after a longer retention interval, which affects memory regardless of set size. We also did a state-trace analysis on the model predictions. The state-trace plot in the lower panel of Fig. 6 shows that the four predicted data points lie on a monotonically increasing line, contrary to the experimental data. This analysis confirms that a unitary memory model with a single latent variable determining performance—here, temporal distinctiveness—cannot explain our findings.

## General discussion

Across two experiments, we examined how a secondary task during retention and PI interact to shape performance in immediate memory tasks that require bindings. By combining these two manipulations within the same experimental designs, we were able to map how the contributions of WM and episodic LTM shift depending on which of them provides more accurate information.

In both experiments, we observed a clear double dissociation between the effects of a secondary task and PI across set sizes. The secondary task impaired performance at smaller set sizes, indicating that WM supports memory when the load is low. In contrast, PI impaired performance at larger set sizes, reflecting greater reliance on episodic LTM once WM capacity is exceeded. We also observed an interaction between the effects of a secondary task and PI. Under conditions of high PI, where retrieval from eLTM is less reliable, a secondary task during retention had a greater impact. This suggests that when episodic LTM is less reliable due to PI, participants are forced to rely more on WM, which is vulnerable to disruption by concurrent processing. Conversely, when PI was low, secondary task effects were restricted to the smallest set sizes, as episodic LTM could effectively support performance under higher load. This pattern supports a dynamic trade-off between WM and LTM: when one system is compromised, the other compensates, if available.

The state-trace analyses provide converging evidence for this two-system interpretation. A purely unitary account predicts that performance at low and high set sizes should vary along a single monotonic function across conditions. Instead, the observed state traces deviated significantly from monotonicity in both experiments. This finding strengthens the conclusion that the effects of PI and secondary-task processing cannot be reduced to variation along one general memory-strength dimension. The comparison with SIMPLE led to the same conclusion: Although the model captured several broad effects of set size, PI, and retention interval, it did not reproduce the critical interaction between set size and secondary-task processing or the non-monotonic state-trace pattern.

Thus, secondary task effects may not emerge in all tasks researchers use to test WM. Whether Brown-Peterson-style tasks reveal secondary task effects likely depends on the extent to which the memory task relies on WM versus LTM. More generally, the present findings suggest that immediate memory performance reflects a flexible mixture of WM and episodic LTM contributions, with their relative involvement determined by memory load and the reliability of the available memory representations, which can be reduced by secondary-task demands and interference.

### Conclusion

Our findings offer converging evidence that performance in immediate memory tasks reflects a flexible mixture of contributions of WM and episodic LTM. Secondary tasks disrupt WM, while PI impairs retrieval from LTM; when both are in play, performance depends on the relative reliability of each system.

## Data Availability

The data can be accessed on the Open Science Framework (https://osf.io/9y3kn/?view_only=c4e737c76ead4a77840fbef6882b53d8).

## Code availability

The analysis scripts can be accessed on the Open Science Framework (https://osf.io/9y3kn/?view_only=c4e737c76ead4a77840fbef6882b53d8)
